# Effects of inorganic phosphate on stem cells isolated from human exfoliated deciduous teeth

**DOI:** 10.1038/s41598-024-75303-6

**Published:** 2024-10-16

**Authors:** Ravipha Suwittayarak, Nunthawan Nowwarote, Chatvadee Kornsuthisopon, Waleerat Sukarawan, Brian L Foster, Hiroshi Egusa, Thanaphum Osathanon

**Affiliations:** 1 Center of Excellence for Dental Stem Cell Biology, Faculty of Dentistry, Chulalongkorn University, 34 Henri-Dunant Road, Wang-Mai, Pathumwan, Bangkok, 10330 Thailand; 2 Department of Oral Biology, Faculty of Dentistry and Reference Center for Skeletal Dysplasia, INSERM UMR1163, Institut Imagine, Necker Hospital, Université Paris Cité, Paris, France; 3 Department of Anatomy, Faculty of Dentistry, Chulalongkorn University, Bangkok, Thailand; 4 Department of Paediatric Dentistry, Faculty of Dentistry, Chulalongkorn University, Bangkok, Thailand; 5 Division of Biosciences, College of Dentistry, The Ohio State University, Columbus, OH USA; 6 Division of Molecular and Regenerative Prosthodontics, Tohoku University Graduate School of Dentistry, Sendai, Miyagi Japan; 7 Division of Advanced Prosthetic Dentistry, Tohoku University Graduate School of Dentistry, Miyagi, Japan

**Keywords:** Stem cells isolated from human exfoliated deciduous teeth, Inorganic phosphate, osteogenic differentiation, Adipogenic differentiation, Osteoblasts, Dental pulp

## Abstract

Calcium phosphate-based materials (CaP) are introduced as potential dental pulp capping materials for deciduous teeth. The present study investigated the influence of inorganic phosphate (P_i_) on regulating stem cells isolated from human exfoliated deciduous teeth (SHED). SHEDs were treated with P_i_. Cell cycle progression and apoptosis were examined using flow cytometry analysis. Osteo/odontogenic and adipogenic differentiation were analyzed using alizarin red S and oil red O staining, respectively. The mRNA expression profile was investigated using a high-throughput RNA sequencing technique. P_i_ increased the late apoptotic cell population while cell cycle progression was not altered. P_i_ upregulated osteo/odontoblastic gene expression and enhanced calcium deposition. P_i_-induced mineralization was reversed by pretreatment of cells with Foscarnet, or p38 inhibitor. P_i_ treatment inhibited adipogenic differentiation as determined by decreased *PPARγ* expression and reduced intracellular lipid accumulation. Bioinformatic analysis of gene expression profiles demonstrated several involved pathways, including PI3K/AKT, MAPK, EGFR, and VEGF signaling. In conclusion, P_i_ enhanced osteo/odontogenic but inhibited adipogenic differentiation in SHED.

**Supplementary Information:**

The online version contains supplementary material available at 10.1038/s41598-024-75303-6.

## Introduction

In dental pulp tissues, stem and progenitor cells are essential regulators of pulpal tissues in response to pulpal inflammation, leading to healing/regeneration processes. When irritation or trauma occurs, stem cells in dental pulp tissues migrate to the injured site and differentiate into odontoblast-like cells, which form reparative dentin^1,2^. Applying pulp-capping materials during a restorative operation stimulates dental stem cell responses and provides an appropriate microenvironment to initiate pulp-dentin tissue regeneration and repair.

Various pulp capping materials have been developed for use in current dental practice, such as calcium hydroxide (CH), mineral tricalcium aggregate (MTA), and calcium silicate-based materials^3,4^. Recently, alternative materials like calcium phosphate-based materials (CaP) have been introduced as potential pulp-capping materials. CaP has also been proposed in several treatments, such as pulpotomy and dental pulp capping, as it can trigger reparative dentin formation^5–7^. The key active components of CaP are calcium ions (Ca^2+^) and inorganic phosphate (P_i_); these ions are the building blocks of hydroxyapatite mineral and play essential roles in the formation, maturation, repair, and regeneration of hard tissues such as bone and dentin^8–12^.

Accumulating evidence over the last two decades supports P_i_ as more than an ionic component of hydroxyapatite in mineralized tissues. P_i_ acts as a signaling molecule for many types of mineralizing cells and their progenitors. P_i_ alters the expression of differentiation and mineralization-associated genes in mouse MC3T3-E1 pre-osteoblasts^13^, mesenchymal stem cells (MSC)^14^, dental pulp stem cells (DPSC)^15,16^, and stem cells isolated from human exfoliated deciduous teeth (SHED)^17^. Evidence that P_i_ can inhibit osteoclastogenesis further implies a role in bone remodeling^18^, and positive effects of P_i_ on M2 macrophage polarization suggest an inhibitory role in inflammation^19^. Inherited and acquired disorders that reduce circulating P_i_ levels, such as nutritional rickets, X-linked hypophosphatemia (XLH), and vitamin D metabolism disorders, cause profound defects in odontoblast function and dentin mineralization^20^. XLH predisposes to dentin hypomineralization, tooth fracture, pulp necrosis, and abscesses^21^.

Because of its cell signaling capabilities and pro-mineralization properties, P_i_ has been considered a potential bioactive agent to enhance pulp-dentin regeneration in regenerative endodontics^15–17^. However, the capability of P_i_ to regulate SHED cell functions remains unclear and further experiments are necessary to support translational and clinical studies on pulp-dentin regeneration. We investigated the effects of P_i_ on SHED cell behaviors, including cell proliferation, migration, and differentiation. Underlying regulatory mechanisms were interrogated by RNA sequencing analysis.

## Results

### Isolation and confirmation of SHED cells

**Fig. 1:**
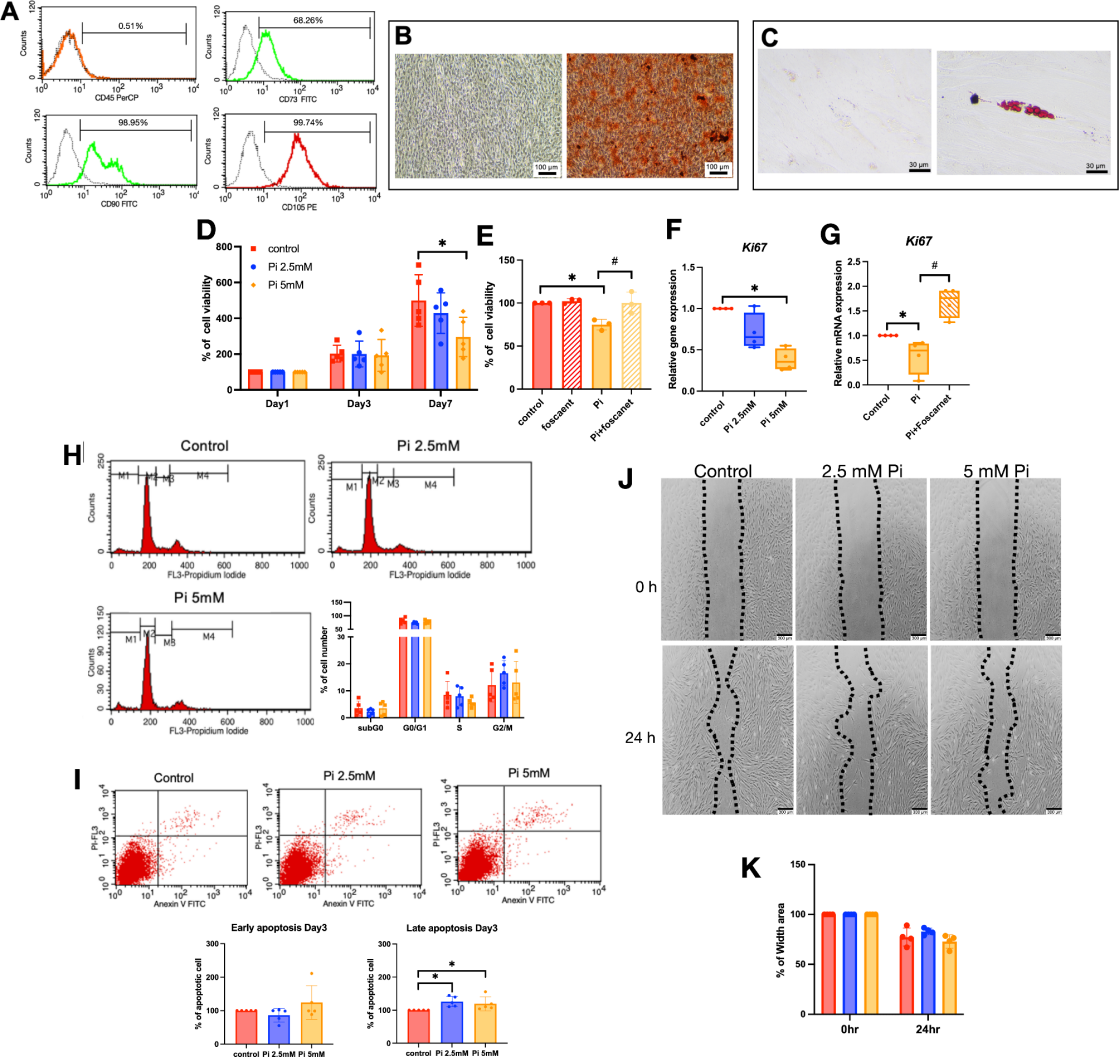
SHEDs characterization and effects of inorganic phosphate on cell functions. CD45, CD73, CD90, and CD105 expression were examined using flow cytometry (**A**). For differentiation ability assay, cells were maintained in an osteogenic or adipogenic induction medium. Mineral deposition and intracellular lipid accumulation were evaluated using Alizarin Red S staining on day 14 (**B**) and Oil Red O staining on day 20 (**C**), respectively. Cells cultured in a normal growth medium were used as the control (left panels). Cells were treated with 2.5 or 5 mM inorganic phosphate (P_i_) for 1, 3, and 7 days. Cell viability was determined using MTT assay (**D**). Cells were treated with foscarnet for 30 min prior to P_i_ exposure and cell viability was examined using the MTT assay on day 7 (**E**). The mRNA expression of *Ki67* was determined at 24 h after P_i_ exposure using real-time polymerase chain reaction (**F** and **G**). On day 3, cells were stained with propidium iodide to determine cell cycle progression by flow cytometry, and the quantitative measurement of proliferative cells was illustrated (**H**). Cells were stained with annexin V/ propidium iodide and further analyzed using flow cytometry. In order to elucidate cell apoptotic numbers, the percentage of cell death was quantified (**I**). Cell migration was examined using an in vitro scratch assay (**J**) and the percentage of area closure was calculated (K). ^*^P < 0.05 compared to the control, ^#^*P* < 0.05 compared to the P_i_ group.

Isolated human dental pulp cells were characterized for mesenchymal stem cell characteristics. Cells expressed CD73, CD90, and CD105, while CD45 was not expressed (Fig. 1A). Under appropriate conditions, cells were able to differentiate into osteoblast-like cells and adipocyte-like cells (Fig. 1B and C). Increased calcium deposition occurred under osteogenic induction conditions (Fig. 1B). Furthermore, adipogenic induction medium promoted lipid droplet formation in the cytoplasm (Fig. 1C). These findings confirm that the isolated cells exhibited dental mesenchymal stem cell characteristics consistent with those reported for SHED cells^22,23^.

## Effects of inorganic phosphate on SHED cell proliferation and cell death

Based on criteria established in previous studies, P_i_ levels used in the investigation were in a range of 1–8 mM^17,24^. Exogenous Pi was required at concentrations higher than 2mM, up to 10mM, to modulate cellular behaviors^14,25,26^. However, a report indicated that at least 4 to 6 mM Pi was required for optimal regulation of osteogenic potential in mesenchymal stem cells (MSC)^14^. Our previous report indicated that 5mM Pi was responsible for enhancing the osteogenic potential of SHED compared to controls receiving no additional P_i_^17^. Here, the minimal and optimal requirements of 2.5 and 5 mM Pi were chosen to elucidate the cellular behaviors of SHED. Cell viability/proliferation was assessed using an MTT assay. On days 1 and 3, there were no differences in the number of viable cells between the 2.5 and 5 mM P_i_ groups. However, by day 7, treatment with 5 mM P_i_ resulted in fewer viable cells (Fig. 1D). To assess whether P_i_ import was required for effects on proliferation, cells were incubated for 30 min with foscarnet (Fos), a sodium-phosphate cotransporter inhibitor. Foscarnet pretreatment reversed the inhibitory effect of P_i_ on cell proliferation at day 7 (Fig. 1E). Expression of *Ki67*, a marker for cell proliferation, was significantly downregulated by 5 mM P_i_, compared with the control at 24 h after treatment (Fig. 1F). Foscarnet pretreatment rescued the P_i_-attenuated *Ki67* expression in SHED cells (Fig. 1G). The cell cycle analysis was performed on day 3. The results demonstrate no differences in cell number in the G0/G1 phase between the control and P_i_ treatment groups. The 5 mM Pi treatment group slightly decreased the S phase population. However, there was no significant difference compared with the control (Fig. 1H).

Apoptosis was next assessed in SHED cells by phosphatidylserine exposure, followed by an assessment of membrane permeabilization by flow cytometry^27,28^. Early and late apoptotic cell numbers were quantified (Fig. 1I). No differences in early apoptotic cell numbers were induced by either 2.5 or 5 mM P_i_ groups, compared to the control. However, both 2.5 and 5 mM P_i_ treated groups significantly increased numbers of late apoptotic cells compared to control.

## Inorganic phosphate does not alter SHED cell migration ability

The in vitro scratch assay was performed to analyze cell migration. A linear gap was created through a confluent layer of SHED cells at the center of tissue culture wells. Cells were cultured in serum-free culture medium to restrain cell proliferation^29^. Cells were then treated with 2.5 or 5 mM P_i_. At 24 h, the width of the scratch area decreased to 77.33% in the control condition, while 2.5 and 5 mM P_i_ treatments showed reduced gap areas of 82.01% and 73.44%, respectively. However, there were no statistically significant differences among groups (Fig. 1J and K).

## Inorganic phosphate promotes osteo/odontoblastic differentiation in SHED cells

**Fig. 2:**
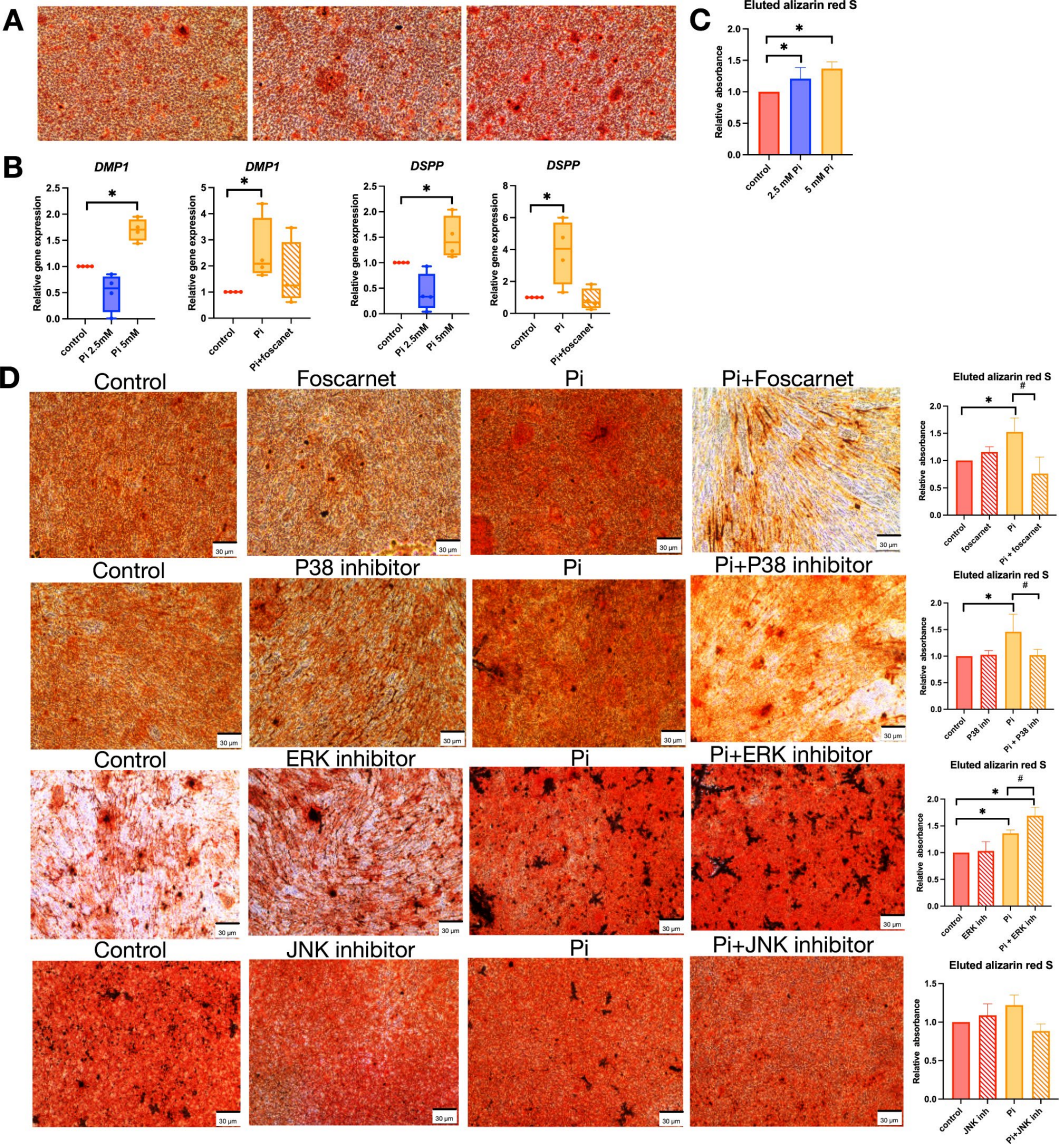
Inorganic phosphate induces mineralization by SHEDs. Cells were treated with 2.5 and 5 mM inorganic phosphate (P_i_) and maintained in an osteogenic induction medium containing P_i_ for 14 days. Mineral deposition was measured using Alizarin Red S staining (**A**). The graph demonstrates the absorbance of eluted dye at 570 nm and normalized to the control (**B**). *DMP1* and *DSPP* mRNA expression levels were assayed using real-time polymerase chain reaction at day 7 (**C**). Cells were pretreated with foscarnet, p38 inhibitor, ERK inhibitor, or JNK inhibitor for 30 min prior to Pi exposure. Cells were maintained in an osteogenic induction medium for 14 days. The mineral deposition was measured using Alizarin Red S staining (**D**). The graph shows the absorbance of eluted dye measured at 570 nm and normalized to the control. ^*^P < 0.05 compared to the control.

Previous studies revealed that P_i_ enhanced the osteogenic potential of MC3T3-E1 pre-osteoblasts^13,18^, DPSC^16^, and MSC^14^. SHED cells underwent an osteogenic induction in the presence of P_i_ to determine effects on in vitro mineralization capability. Addition of either 2.5 or 5 mM P_i_ increased mineral deposition compared to controls (Fig. 2A and C). Compared to controls, 5 mM P_i_ enhanced mRNA expression of *DMP1* and *DSPP*, odontoblast-expressed genes encoding proteins essential for dentin mineralization (Fig. 2B). This phenomenon was attenuated by pre-treated cells with foscarnet (Fig. 2B).

To investigate the potential mechanisms for regulation of mineralization, cells were pretreated with foscarnet, p38 inhibitor, ERK inhibitor, or JNK inhibitor for 30 min prior to P_i_ exposure. Cells were then maintained in osteogenic induction for 14 days in the presence of P_i_ and inhibitors. P_i_-induced mineralization was attenuated when cells were treated with foscarnet or p38 inhibitor, but not in the presence of ERK or JNK inhibitors (Fig. 2D). Interestingly, the ERK inhibitor promoted increased mineralization compared to the controls.

## Inorganic phosphate attenuates adipogenic differentiation of SHED cells

**Fig. 3:**
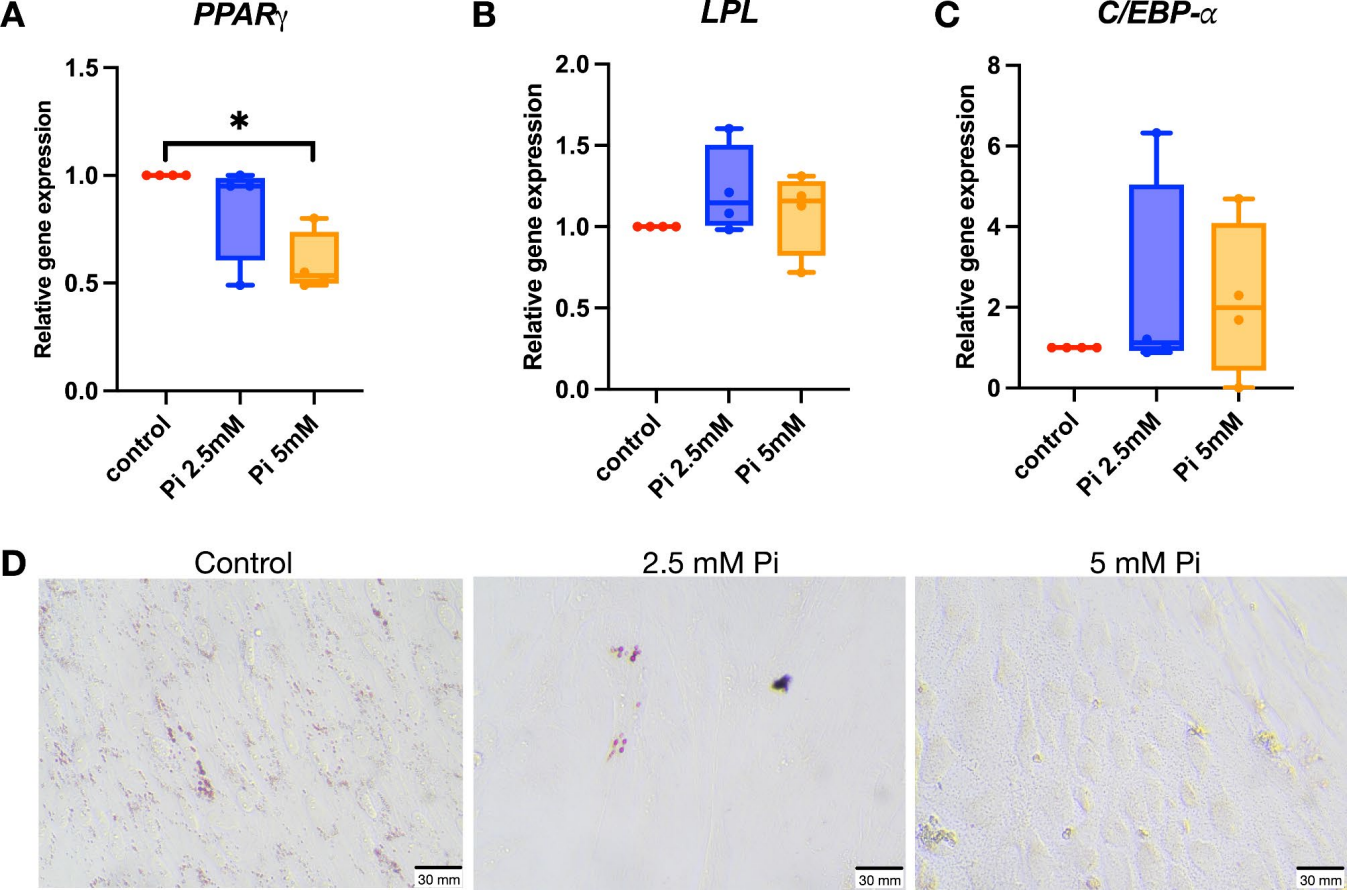
Inorganic phosphate attenuates adipocyte differentiation of SHEDs. Cells were treated with 2.5 and 5 mM P_i_ and maintained in adipogenic induction medium. *PPAR-γ*, *C/EBP-α*, and *LPL* mRNA expression levels were analyzed on day 8 using real-time polymerase chain reaction (A-C). The intracellular lipid accumulation was determined using Oil Red O staining at day 20 (D). ^*^P < 0.05 compared to the control.

Increased P_i_ appeared to increase the expression of odontoblast markers and in vitro mineral deposition by SHED cells. We tested the effects of Pi on adipogenic differentiation to better understand the impact on cell fate. SHED cells were incubated in an adipogenic induction medium in the presence of 2.5 and 5 mM P_i_. Adipocyte differentiation-related genes, including *PPARγ*, *LPL*, and *C/EBP-α*, were analyzed on day 8. *PPARγ* mRNA levels were significantly downregulated by 5 mM P_i_ treatment compared to controls (Fig. 3A), though P_i_ did not affect *LPL* and *C/EBP-α* mRNA expression (Fig. 3B and C). Intracellular lipid accumulation was observed using Oil Red O staining at day 20. Reduced lipid accumulation was noted under P_i_ treatment (Fig. 3D).

### Differential transcriptomic profiling after induction of pi

**Fig. 4:**
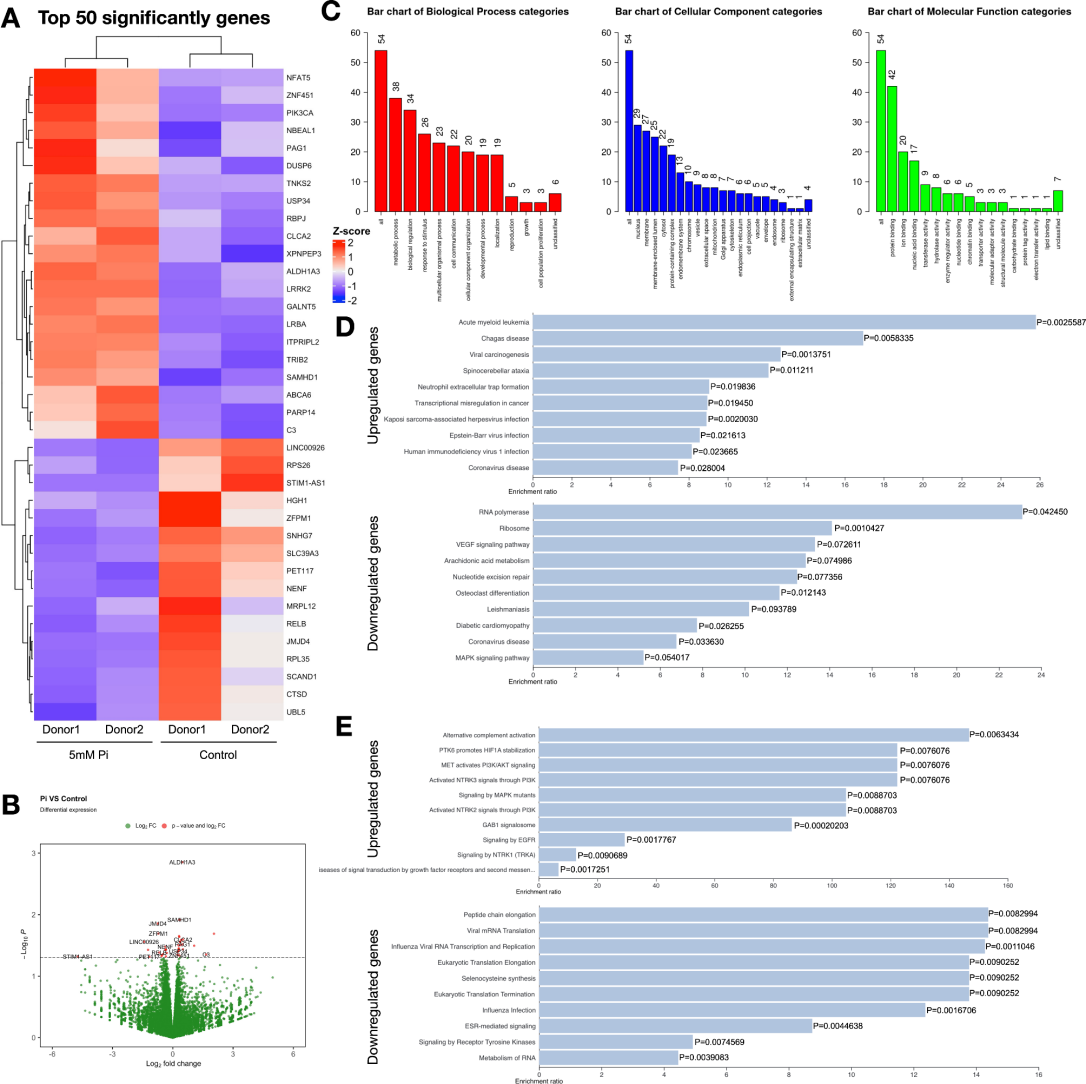
Transcriptomic profile of inorganic phosphate-treated SHEDs. Cells were treated with 5 mM P_i_ for 24 h. RNA was extracted and subjected to RNA sequencing analysis for differential gene expression. The top 50 significantly expressed genes were included in the heat map (**A**). The distribution of all significantly differential genes was shown in the volcano plot (**B**). Differentially expressed genes were investigated in GO term analysis (**C**). Functional and pathway enrichments were analyzed in KEGG (**D**) and Reactome pathway database (**E**). Control1 and Control2 as representatives for Control condition from 2 different donors, Pi1 and Pi2 as representatives for 5mM Pi treated conditions from 2 different donors, ^*^P < 0.05 compared to the control.

Selective gene analyses revealed P_i_ regulates odontoblastic and adipogenic genes in SHED cells. In order to identify underlying regulatory mechanisms, SHED cells were treated with 5 mM P_i_ and total RNA was collected and subjected to RNA sequencing analysis for differential mRNA expression profiling. The top 50 differentially expressed genes identified from P_i_ treatment were illustrated as a heat map (Fig. 4A). Volcano plots demonstrated the distribution of expressed genes (Fig. 4C). Gene ontology analysis of the differentially expressed genes was enriched for biological process (red bar), cellular component (blue bar), and molecular function (green bar) (Fig. 4C).

From pathway enrichment analysis by KEGG, The significant genes were identified to be enriched in the VEGF signaling pathway and MAPK signaling pathway (Fig. 4D). For Reactome enrichment analysis, the upregulated genes were enriched in several pathways (Fig. 4E), including PTK6 promotes HIF1A stabilization, MET activates PI3K/AKT signaling, activated NTRK3 signals through PI3K, while the downregulated genes were organized in pathways, including ESR-mediated signaling, signaling by receptor tyrosine kinase (Fig. 4E).

## Discussion

P_i_ is a free form of phosphorus complex existing in extracellular fluid^9^ and plays essential roles in bone and tooth development^30,31^. P_i_ concentrations are regulated at both the systemic and local levels. Systemically, the gut-kidney-bone axis controls circulating P_i_ levels through endocrine mechanisms employing vitamin D, parathyroid hormone (PTH), and fibroblast growth factor 23 (FGF23)^20,32^. The normal circulating range of P_i_ typically stays within 0.5–1.5 mM^33^. At the local cell and tissue levels, P_i_ levels are controlled by a host of enzymes and transporters that work cooperatively and antagonistically with one another. Tissue non-specific alkaline phosphatase (TNAP) is a cell membrane-bound enzyme that generates P_i_ ions from inorganic pyrophosphate and other phosphorylated substrates^34^. Orphan phosphatase 1 (PHOSPHO1) generates P_i_ within the confines of matrix vesicles, which promotes the initiation of mineralization in some calcified tissues^35^. P_i_ transporters regulate the import of Pi into cells and play roles in the sensing and signaling capabilities of P_i_ in mineralized tissue cells^36^. Dysregulation of either systemic or local P_i_ metabolism results in dramatic effects on cell function and tissue mineralization^21,32,37^.

P_i_ was shown in prior publications to induce signaling in a range of mineralized tissue cells^13,25,38–40^. In the present study, we investigated effects of P_i_ at concentrations of 2.5 and 5 mM, consistent with the range of previous publications^14,16,17,26^. While some cell populations were reported to have sensitivity to high P_i_^41,42^, these concentrations were non-toxic to SHED cells. Similarly, a recent report demonstrates that mesenchymal stem cells survive in the presence of 10 mM Pi^14^.

In addition to being a regulator of mineralization, P_i_ is necessary for numerous cell functions, including cell growth and proliferation, through the phosphorylation of intracellular enzymes. In non-mineralizing cells, the increased extracellular P_i_ levels promoted P_i_ influx into the cell, resulting in increased cell proliferation^43^. P_i_ activates AKT phosphorylation and the ERK signaling cascade, leading to the enhancement of cell proliferation^44^. Some studies indicate that PiT1, a sodium-P_i_ co-transporter, participates in the enhancement of cell proliferation. *PiT1* silencing decreased P_i_ influx, leading to decreased HeLa cell proliferation. However, cell proliferation inhibition of *PiT1* silencing does not depend on the P_i_ transport function; the functional deletion of the P_i_ transporter activated the P38 signaling pathway^45,46^.

The present study demonstrated that SHED cell number was decreased in P_i_-treated groups at day 7, compared with the control. Furthermore, reduction of *Ki67* expression was also noted in the 5 mM P_i_-treated cells. These effects were rescued when cells were pretreated with foscarnet, an inhibitor of P_i_ transport. However, there was no significant difference in the S or G2/M populations in those cells treated with P_i_, compared with the control. Consistent with mineralizing cell, the impairment of proliferative activity in cells exposed to P_i_ has previously been reported in such as osteoblast and chondroblast^24,41^. Further, our present study found the influence of Pi on cell apoptosis. P_i_-induced mineralizing cell apoptosis occurs during the resorptive activity of osteoclasts, leading to the release of P_i_^47^. Mechanistically, P_i_-induced bone cell apoptosis promotes Ca^2+^ influx, resulting in mitochondria dysfunction and increasing levels of reactive oxygen species (ROS)^47–50^.

Our results showed that P_i_ promoted mineral deposition and induced *DSPP* and *DMP1* mRNA expression in SHED, similar to previous reports^16,51^. *DMP1* and *DSSP* have been used as markers for odonto/osteogenic differentiation^52,53^. The increased mineralization seen in the present study may not be from the direct precipitation of the binding between phosphate ions and calcium ions available in the medium. Previous reports demonstrated that culture medium supplemented with inorganic phosphate did not show increased mineralization in the condition without cells. In this regard, adding up to 10 mM Pi in the culture medium did not mark an increase in alizarin red S staining in the condition without cells^14^. This evidence implies that the significant increase of mineralization upon treated cells with inorganic phosphate occurred via biological processes. This observation was noted in other publications on other cell types^14,54^. However, several biological hypotheses need further clarification, for example, the possible calcium depletion effects on cell responses and the change of cell behaviors due to the potential apoptotic effect of Pi.

This finding supports that P_i_ induces odonto/osteogenic differentiation in SHED. The mitogen-activated protein kinases (MAPKs) signaling is indeed one of remarkable pathway for growth factor-induced osteogenic differentiation^55^. A member family of MAPK, including extracellular signal-related kinases (ERKs), c-Jun N terminal kinases (JNKs), and P38 can be activated by MAP3K, upstream MAPK activator, phosphorylating MAPK family^56^.

As for Pi activation-regulated pathways, Pi induces the ERK1/2 signaling pathway in the upregulation of OPN, matrix Gal protein, and DMP1 by MC3T3-E1 pre-osteoblasts and dental pulp stem cells (DPSC)^13,16^. Our study demonstrated that P38 inhibition attenuated the effects of Pi-induced mineralization in SHEDs. P38 was shown to be part of osteogenic differentiation inductive pathways in mesenchymal stem cells^57^. ERK and JNK inhibitors did not reverse Pi-induced mineralization, whereas ERK inhibition enhanced Pi-induced mineralization in SHED, consistent with previous study^58^. Since ERKs and P38 pathways are a downstream of MAP3K, we speculate that the ERK inhibition may increase P38 activation, resulting in the enhancement of mineral deposition in SHEDs by an increase of P38 activation. Here, this finding demonstrated that Pi is responsible for promoting mineral deposition in SHEDs though P38 and ERK signalling pathway. However, the further study on targeting to P38 and ERK pathway to enhance osteogenic potential in SHEDs needed investigation.

During the adipogenic differentiation process, MSC expresses specific gene markers, including *PPAR-γ*, *C/EBP-α*, and *LPL*^59^. In our study, P_i_ treatment inhibited the expression of *PPAR-γ* and reduced intracellular lipid droplet formation. A previous study found that adipogenic potential was regulated by the MAPK family. ERK and JNK signaling have been demonstrated as activators, while P38 signaling has been shown to inhibit adipogenesis^60^. This finding implicates that, in the presence of elevated P_i_, SHED cells are induced to commit toward osteogenic differentiation rather than adipogenic differentiation.

We used transcriptomic analysis to further investigate signalling effects of P_i_ on SHED cell differentiation. The expression of *RELB* gene was downregulated in Pi treated group. A recent report has showed that abolished expression of RelB have promoted cell apoptosis^61^ and inhibited cell proliferation^62^, similar to our finding. Additionally, Reactome enrichment analysis showed that significantly upregulated genes were enriched in signaling by MAPK mutant, which is relevant to DUSP6. DUSP6 is well-recognized as MKP3, a specific phosphatase for ERK signaling in skeleton development^63^. Additionally, DUSP6 has played an essential role in maintaining phosphorylated P38 signaling^64^. A study has revealed that the DUSP6 depletion in mouse models caused dwarf-consisted skeleton abnormalities^65^. In this regard, inducible DUSP6 expression has been reported to inhibit cell proliferation in hair follicle stem cells^64^ and induce cell apoptosis^66^. These findings were consistent with the in vitro investigation demonstrating that P_i_ promoted mineralization via the P38 signaling pathway but induced apoptosis in SHED.

As for dental materials, P_i_ is based on most pulp capping materials, along with calcium. Less Pi release may not be enough to influence neighboring cells. Recently, calcium phosphate-based material, an alternative dental material, can provide persistently calcium and phosphate ions^8–12^. Both calcium and phosphate ions were reported to be key modulators for osteogenic differentiation. Consistent with our observation, Pi was shown to have influences in the induction of early-stage osteogenic inducer genes, for example, the *DMP1* gene^67^. On the contrary, calcium ion has a role in promotion of late-stage osteogenic genes^68,69^. We speculate that the presence of both calcium and phosphate ions may function in different steps of tissue mineralization and also accelerate mineralized tissue repair.

Mesenchymal stem cells (MSC) possess the self-renewal and multipotential differentiation ability. Numerous surface markers have been proposed to identify the MSC population, including CD105, CD90, CD73, CD44, CD166, CD29, STRO-1, CD146, and CD271^70^. However, the International Society for Cellular Therapy set three minimum criteria to identify MSC^22^. First, MSC must be the adherent cells on tissue culture surfaces. Second, MSC must express CD105, CD73, and CD90, and lack expression of CD45, CD34, CD14 or CD11b, CD79α or CD19, and HLA-DR surface molecules. Third, MSC must possess multipotential differentiation. In the present study, we evaluated the expression of CD105, CD90, and CD73, which are considered the minimum and common MSC surface markers^70^. In addition, we demonstrated the mineral deposition and intracellular lipid accumulation ability after cultured in osteogenic and adipogenic induction medium, respectively. Together, our data illustrated the MSC characteristics of the cells isolated from the remaining dental pulp tissues of the exfoliated deciduous teeth.

**Fig. 5:**
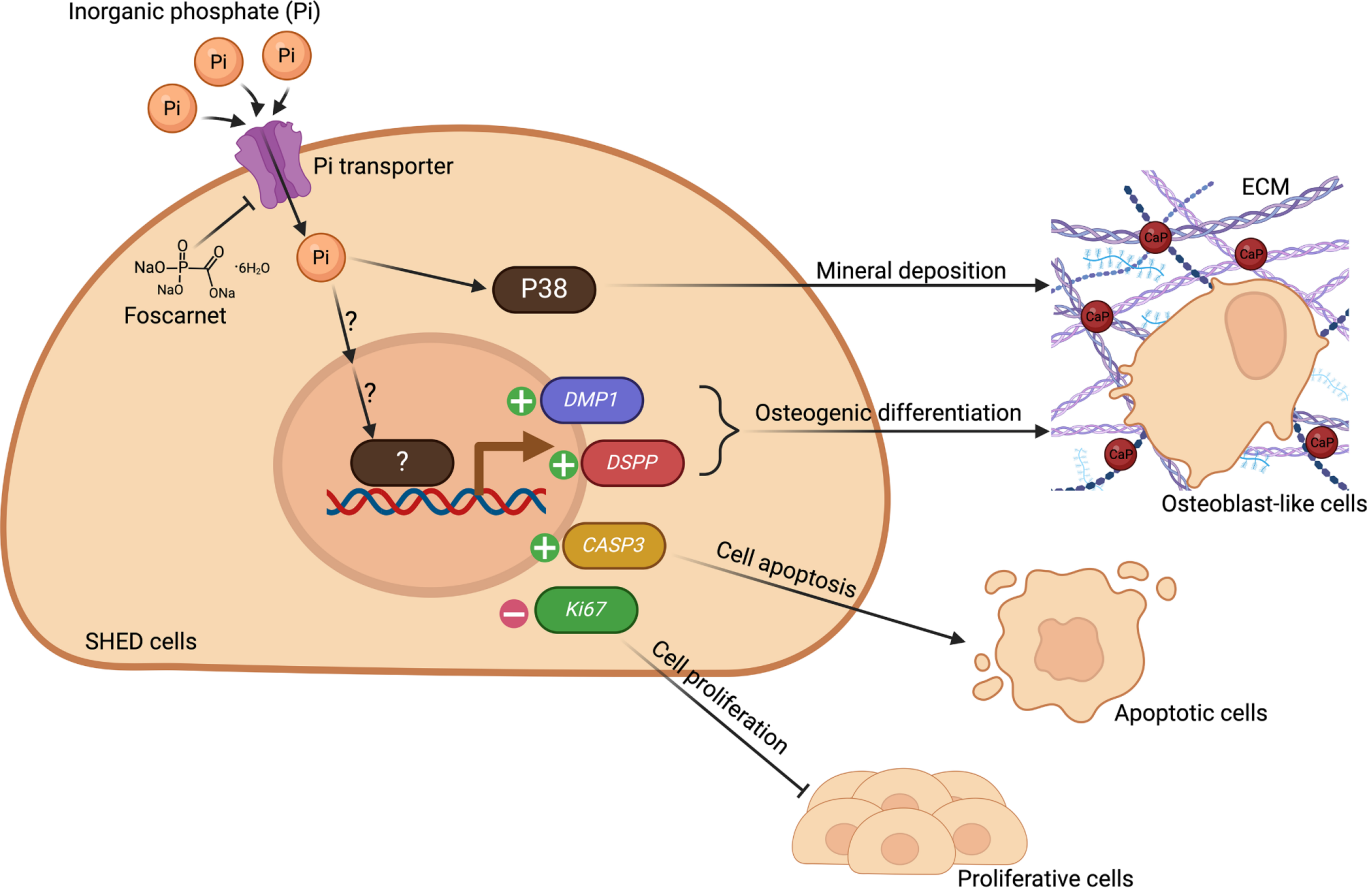
Schematic diagram of inorganic phosphate’s effects on regenerative responses and multipotency of SHEDs. The presence of P_i_ regulated SHED cell number via the promotion of cell apoptosis. Additionally, P_i_ reduced the *Ki67* gene expression. P_i_ induced the gene expression of *DMP1* and *DSPP* to undergo osteogenic induction. Also, P_i_ enhanced the osteogenic potential of SHEDs via the p38 pathway. Created with Biorender.com.

In summary, our data derived from in vitro experiments and mRNA expression profiles demonstrated strong evidence that P_i_ regulates cell proliferation and differentiation via different mechanisms in SHEDs. The present study reveals the effects of P_i_ induced on the osteo/odontogenic ability of SHEDs via the P38 signaling (Fig. 5). Our finding contributes insight into the mechanism of P_i_ on SHED in regulating regenerative potentials. Additional experiments are needed to investigate translational implications and clinical potential for P_i_ to positively promote SHED cell differentiation and tertiary dentin formation after dental procedures.

## Materials & methods

### Cell isolation and culture

The remaining dental pulp tissues from the exfoliated primary teeth were collected from healthy donors scheduled for extraction according to the clinical treatment plan from the Department of Paediatric Dentistry, Faculty of Dentistry, Chulalongkorn University (Bangkok, Thailand). The protocol was approved by the Human Ethical Research Committee, Faculty of Dentistry, Chulalongkorn University (No. 131/2023). The written informed consent was obtained. Methods were carried out in accordance with the Declaration of Helsinki. Cell isolation was performed using explantation protocol^17^. The migrated cells were cultured in the high glucose Dulbecco’s Modified Eagle Medium (DMEM) supplemented with 10% fetal bovine serum (FBS), 1% L-glutamine (2 mM), 1% of penicillin (100 U/mL), streptomycin (100 mg/mL) and incubated at 37 °C in a humidified 5% CO_2_ atmosphere. DMEM contained sodium phosphate monobasic at the concentration of 0.9 mM and a sodium bicarbonate buffer system. The culture medium was changed every 48 h. When the cells reached confluence, they were subcultured at a 1:3 ratio using 0.25% trypsin/EDTA (Gibco). Cells in passages 3–7 were used in the experiments.

To prepare inorganic phosphate, the 500 mM sodium phosphate (Na_2_HPO_4_, Sigma-Aldrich) was prepared and produced to 500 mM inorganic phosphate. Cells were treated with 2.5 or 5 mM inorganic phosphate, according to our previous study^17^. The media was replaced with fresh media containing inorganic phosphate P_i_ every 2 days. For the inhibition experiment, cells were treated with 0.1 mM Foscarnet (CAS No. 34156-56-4, Sigma-Aldrich), 10 µM ERK inhibitor (CAS No. 1049738-54-6, Sigma-Aldrich), 10 µM JNK inhibitor (SP600125, CAS No. 129-56-6, Sigma-Aldrich), or 1 µM p38 inhibitor (SB239063, CAS No. 193551-21-2, Sigma-Aldrich) for 30 min before P_i_ treatment.

### Alizarin red S staining

The cells were seeded in a 24-well plate at a density of 25,000 cells per well and cultured in an osteogenic medium containing 10% FBS-DMEM supplemented with 50 µg/mL L-ascorbic acid, 100 nM dexamethasone and 5 mM β-glycerophosphate. The medium was changed every two days. On day 14, cells were washed with deionized water and fixed with cold methanol. Mineral deposition was stained with an Alizarin Red S solution at room temperature with gentle agitation. Unbound dye was removed by washing with deionized water. Images of calcium nodules were captured with an inverted microscope. The staining samples were dissolved in 10% cetylpyridinium chloride monohydrate in 10 mM sodium phosphate under gentle agitation^71^. The solution was subjected to absorbance measurement at 570 nm using a microplate reader (Biotek ELX800, USA).

### Oil red O staining

Cells were cultured for 20 days in an adipogenic medium consisting of 10% FBS-DMEM supplemented with 500 µM 3-Isobuty-1-methylxanthine (IBMX), 1 µg/mL insulin from bovine pancreas, and 100 µM indomethacin. The medium was changed every four days. Cells were fixed with 10% buffered formalin and then stained with Oil Red O solution. Lipid droplets were visualized under an inverted microscope.

### MTT assay

The cells were seeded in a 24-well plate at a density of 12,500 cells per well and cultured in a normal growth medium for 1, 3, and 7 days. MTT solution was added to each well for 30 min to determine mitochondrial enzyme metabolism. The insoluble formazan was eluted by DMSO and subsequently subjected to measuring the absorbance at 460 nm using a microplate reader, according to the manufacturer’s protocol. The percentage of viable cells was calculated from these data.

### Real-time RT-PCR

Total cellular RNA was extracted by using RiboExTM solution. RNA quality and concentration were measured using Nanodrop (Thermo Scientific, USA). RNA was converted into complementary DNA using an ImProm-IITM Reverse Transcription System. One microliter of complementary DNA was used for real-time polymerase chain reaction using a FastStart Essential DNA Green Master kit. The reaction was performed on a Bio-Rad PCR system. Relative gene expression was calculated using the 2^−ΔΔCt^ method^72^. Expression values of target genes were normalized to the *GAPDH* expression values and the control. The oligonucleotide primers used for this study were as follow; *GAPDH* forward 5’-CACTGCCAACGTGTCAGTGGTG-3’, reverse 5’- GTAGCCCAGGATGCCCTTGAG − 3’; *Ki67* forward 5’-CGTTTGTTTCCCCAGTGTCT-3’, reverse 5’- CTCCCTGCCCCTTTCTATTC-3’; *DMP1* forward 5’- CAGGAGCACAGGAAAAGGAG-3’, reverse 5’- CTGGTGGTATCTTGGGCACT-3’; *DSPP* forward 5’-CAACCATAGAGAAAGCAAACGCG-3’, reverse 5’-TTTCTGTTGCCACTGCTGCTGGAC − 3’; *PPAR-γ* forward 5’-CCAGTGGTTGCAGATTACAAGTATG − 3’, reverse 5’- TTGTAGAGCTGAGTCTTCTCAGAATAATAAG-3’; *LPL* forward 5’-GAGATTTCTCTGTATGGCACC-3’, reverse 5’-CTGCAAATGAGACACTTTCTC − 3’; C/EBP-alpha forward 5’-CGGTGGACAAGAACAGCAAC-3’, reverse 5’-CGGAATCTCCTAGTCCTGGC-3’.

### Apoptosis assay

Cells were detached from the well using trypsin and centrifuged at 5,000 rpm for 5 min, and then the supernatant was discarded. Cells were suspended with 400 µL of PBS. 50 ng/mL of propidium iodide (PI) was added to each tube, followed by 5 µL of annexin V reagent. After dark incubation for 15 min, 100 µL of annexin V buffer was added and mixed gently. The stained cells were analyzed by flow cytometry. Viable cells gated cell size and granular content were used to determine the phenotype of cell apoptosis.

### Cell cycle analysis

Cells were detached from the well using trypsin and centrifuged at 5,000 rpm for 5 min to collect cell palate. The palate was suspended with 400 µL of 70% ethanol for 15 min, then was washed with PBS and rinsed the supernatant. Cells were incubated with 500 µL PBS containing 40 µg/mL of RNase for 30 min. Finally, cells were stained with 50 ng/mL of PI prior to measuring by flow cytometry. The viable cells were only gated to determine cell cycle progression by determining cell size and granular content.

### Cell migration

Cells were seeded to be confluent and maintained in a growth medium for 24 h. The scratch line was created with a sterile pipette tip. The image of cells was taken using an inverted microscope at 0, 24, and 48 h at the same reference area. The width of the scratch area was measured using ImageJ software.

### RNA sequencing

The mRNA profile was determined using the NextSeq 5000 desktop sequencing system at the Omics Sciences and Bioinformatics Center (Faculty of Science, Chulalongkorn University). The cells were treated with 5 mM P_i_ for 24 h in normal growth medium. Subsequently, RNA was isolated using RNeasy kit (Cat. No. 74104, Qiagen, MD, USA). DNase treatment was performed in columns. Total RNA quantity and quality were examined using a Nanodrop and Aligent 2100 Bioanalyzer system. Library quality assurance was conducted using an Aligent 2100 Bioanalyzer system and Qubit 3.0 fluorometer. Sequencing was performed in a NextSeq 500 sequencing system. Read quality was checked, trimmed, and filtered with a FastQC and FastX toolkit^73^. Reads were mapped with *Homo sapiens* UCSC hg38 using TopHat2^74,75^. FPKM estimation of reference genes and transcripts was performed using DeSeq2 analysis. The differentially expressed genes were further analyzed for pathway enrichment using network-based visual analytics for gene expression profiling, meta-analysis, and interpretation, NetworkAnalyst^76^. Sequencing data were submitted to the NCBI’s Gene Expression Omnibus (GSE266257).

### Statistical analysis

Data are reported as mean ± standard deviation. Statistical analyses were evaluated using the Man-Whitney U test or Kruakal-Wallis test, followed by pair-wise comparison. *P* < 0.05 was considered statistically significant. The analysis was performed using statistical software (GraphPad Prism 10.2.2). The experiments were performed with at least four biological replicates (*n* = 4). For RNA sequencing analysis, the samples were evaluated from 2 different donors (*n* = 2).

## Electronic supplementary material

Below is the link to the electronic supplementary material.

Supplementary Material 1

## Data Availability

The datasets generated during and/or analyzed during the current study are available in the NCBI’s Gene Expression Omnibus (GSE266257). All data generated during and/or analyzed during the current study are available from the corresponding author upon reasonable request.
