# Supplementary material for: Effects of inorganic phosphate on stem cells isolated from human exfoliated deciduous teeth
Source: Sci Rep. 2024 Oct 16;14:24282. doi: 10.1038/s41598-024-75303-6 (PMC11484878; doi:10.1038/s41598-024-75303-6)
Supplement: Supplementary file 1 — Supplementary Material 1 [file 41598_2024_75303_MOESM1_ESM.docx]

***Table1.*** Effect of P_i_ on cell cycle of SHEDs

| Cell phases | Control  (average ± S.D.) | 2.5 mM Pi  (average ± S.D.) | 5 mM Pi  (average ± S.D.) |
| --- | --- | --- | --- |
| SubG0 | 3.564 ± 2.58 | 3.37 ± 1.19 | 35.04 ± 2.46 |
| G0/G1 | 81.694 ± 10.21 | 76.42 ± 3.56 | 78.152 ± 6.16 |
| S | 8.504 ± 4.99 | 3.65 ± 3.65 | 5.582 ± 1.63 |
| G2/M | 12.102 ± 6.21 | 16.77 ± 4.70 | 13.08 ± 7.82 |

^*^*P* < 0.05 compared to the control, ^#^*P < 0.05* compared to the P_i_ group.

***Table2.*** Effect of P_i_ in regulation of cell apoptosis

| Condition | fold-change of early apoptotic cell | S.D. | fold-change of Late apoptotic cell | S.D. |
| --- | --- | --- | --- | --- |
| control | 1.00 | ± 0.00 | 1.00 | ± 0.00 |
| 2.5 mM P_i_ | 1.35 | ± 0.66 | 1.18^*^ | ± 0.23 |
| 5 mM P_i_ | 1.25 | ± 0.99 | 1.17^*^ | ± 0.20 |

^*^*P* < 0.05 compared to the control, ^#^*P < 0.05* compared to the P_i_ group.
